# Prominent immune signatures of T cells are specifically associated with indolent B‐cell lymphoproliferative disorders and predict prognosis

**DOI:** 10.1002/cti2.1105

**Published:** 2020-01-22

**Authors:** Shuhua Yi, Yu Zhang, Wenjie Xiong, Weiwei Chen, Zhaohua Hou, Yang Yang, Yuting Yan, Yunbo Wei, Rui Cui, Huijun Wang, Zhen Yu, Heng Li, Zengjun Li, Wei Liu, Rui Lv, Tingyu Wang, Kun Ru, Dehui Zou, Minglei Shu, Lugui Qiu, Di Yu

**Affiliations:** ^1^ State Key Laboratory of Experimental Hematology National Clinical Research Center for Blood Diseases Institute of Hematology and Blood Disease Hospital Chinese Academy of Medical Sciences and Peking Union Medical College Tianjin China; ^2^ Shandong Analysis and Test Center Laboratory of Immunology for Environment and Health Qilu University of Technology (Shandong Academy of Sciences) Jinan China; ^3^ Shandong Artificial Intelligence Institute Qilu University of Technology (Shandong Academy of Sciences) Jinan China; ^4^ Translational Research Institute The University of Queensland Diamantina Institute Brisbane QLD Australia; ^5^ Department of Hematology Tianjin First Center Hospital Tianjin China; ^6^ Department of Immunology and Infectious Disease John Curtin School of Medical Research Australian National University Canberra ACT Australia

**Keywords:** B‐cell lymphoproliferative disorders, indolent, prognosis, T‐cell immunological signature

## Abstract

**Objectives:**

T cells play an essential role in controlling the development of B‐cell lymphoproliferative disorders (BLPDs), but the dysfunction of T cells in BLPDs largely remains elusive.

**Methods:**

Using multiplexed flow cytometry, we quantified all major subsets of CD4^+^ helper T cells (Th) and CD8^+^ cytotoxic T cells (Tc) in 94 BLPD patients and 66 healthy controls. Statistics was utilised to rank T‐cell signatures that distinguished BLPDs from healthy controls and differentially presented between indolent and aggressive categories.

**Results:**

By comparing with healthy controls, we found that the indolent but not aggressive type of BLPDs demonstrated a high degree of T‐cell activation, showing the increase in type I helper T (Th1) cells and follicular B‐helper T (Tfh) cells, both of which strongly associated with the enhanced differentiation of exhaustion‐like effector cytotoxic CD8^+^ T cells expressing PD‐1 (Tc exhaustion‐like) in indolent BLPDs. Random forest modelling selected a module of T‐cell immune signatures best performing binary classification of all BLPD patients. This signature module was composed of low naïve Th cells and high Th1, Tfh and Tc exhaustion‐like cells which efficiently identified > 85% indolent cases and was, therefore, assigned as the Indolent Dominant Module of T‐cell immune signature. In indolent BLPD patients, a strong bias towards such signatures was found to associate with clinical characteristics of worse prognosis.

**Conclusion:**

Our study identified a prominent signature of T‐cell dysregulation specifically for indolent BLPDs, suggesting Th1, Tfh and Tc exhaustion‐like cells represent potential prognostic biomarkers and targets for immunotherapies.

## Graphical abstract

The dysregulated function of the immune system is a major risk factor for B‐cell lymphoproliferative disorders. Here, we systematically characterized the composition of all the major functional subsets of CD4^+^ helper T cells and CD8^+^ cytotoxic T cells in various BLPD subtypes, categorized into indolent and aggressive groups. We identified indolent but not aggressive BLPDs with a prominent T‐cell immune signature of excessive generation of follicular helper T cells and hyperactivation of cytotoxic T cells that are driven towards exhaustion by the expression of PD‐1.

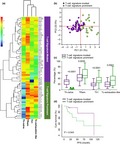

## Introduction

B‐cell lymphoproliferative disorders (BLPDs) are a collection of lymphoid malignancies that are characterised by the abnormal accumulation of B lymphocytes in bone marrow and peripheral lymphoid tissues.^1^ Animal models have convincingly demonstrated that T cells can recognise and eliminate malignant B cells to prevent the development of BLPDs,^2^ suggesting that the dysfunction of the immune system could be a major risk factor for BLPDs. In line with the observation in mouse models, several studies reported altered T‐cell composition and function in BLPD patients.^3^, ^4^, ^5^, ^6^, ^7^, ^8^, ^9^ Although results from human studies were not always consistent and sometimes contradictory, probably due to distinct BLPD types and different criteria for patient recruitment, most studies suggested a breakdown of immune surveillance in BLPDs, demonstrated by excessive regulatory T (Treg) cells^3^, ^4^, ^5^ and the dysfunctional phenotype of CD8^+^ T cells towards exhaustion^6^, ^7^, ^8^, ^9^ in patients. Such findings provided rationales for the application of T‐cell immune signature in the discovery of prognostic markers and the development of targeted immunotherapies. However, it remains challenging to know which types of BLPDs, within such a diversified collection, are more prone to immune dysregulation, and, consequently, should be given a priority for further investigation.

Here, we systematically characterised the composition of all major functional subsets of CD4^+^ helper T cells (Th) and CD8^+^ cytotoxic T cells (Tc) in non‐Hodgkin lymphoma (NHL) BLPD patients. The cohort was composed of both indolent and aggressive categories with various subtypes. We identified indolent but not aggressive BLPDs showing a prominent T‐cell immune signature of the excessive generation of type I helper T (Th1) cells and follicular helper T (Tfh) cells, and the hyperactivation of cytotoxic T cells that are likely driven towards exhaustion by the expression of PD‐1. Therefore, these T‐cell immune signatures represent prior candidates for biomarker discovery and target immunotherapy development, especially in indolent BLPDs.

## Results

### T‐cell signatures are primarily associated with indolent BLPDs

Treatment‐naïve patients with BLPDs (non‐Hodgkin's lymphomas, *n* = 94) were recruited and clinically categorised as indolent (*n* = 75) or aggressive (*n* = 19). The indolent BLPDs included chronic lymphocytic leukaemia (CLL), follicular lymphoma (FL), hairy cell leukaemia (HCL), splenic marginal zone lymphoma (SMZL), nodal marginal zone lymphoma (NMZL), mucosa‐associated lymphoid tissue (MALT) lymphoma, lymphoplasmacytic lymphoma/Waldenström's macroglobulinemia (LPL/WM) and other unclassified chronic BLPDs. While the aggressive BLPDs were composed of diffuse large B‐cell lymphoma (DLBCL), Burkitt lymphoma (BL), mantle cell lymphoma (MCL) and B‐lymphoblastic lymphoma (B‐LBL) (Supplementary table 1). All except one B‐LBL patient were subsequently treated with rituximab‐contained regimens. Patients with indolent BLPDs were significantly older than those with aggressive BLPDs (median age: 56 vs. 47 years old, *P* = 0.0066). As a result, a single healthy control group was unable to match the ages for both patient groups. We, therefore, recruited healthy subjects (25–50 years old, *n* = 66) since the immune system from middle‐aged adults is regarded as the representative for a normal state.

T‐cell function was analysed using flow cytometry to measure all major subsets of CD4^+^ Th cells and CD8^+^ Tc cells (the gating strategy shown in Supplementary figure 1 and the details for all 15 T‐cell immunological signatures listed in Supplementary table 2). Among the total 15 T‐cell signatures (Supplementary figure 2a), both CD4^+^ and CD8^+^ T‐cell frequencies were dramatically reduced in BLPD patients, confirming the pathological expansion of B lymphocytes. Due to the different age ranges among healthy and patient groups, we first identified age‐associated changes of T‐cell immunological signatures so that these potential confounding effects could be excluded for between‐group comparison. In the healthy cohort, we identified an age‐related decline of CD45RA^+^CCR7^+^‐naïve (Th‐naive) population, while a corresponding increase of CD45RA^−^ memory (Th‐M) cells in Th cells (Supplementary figures 2b and 3), in line with reports showing increased immune activation with age.^10^ Similarly, patients with indolent BLPDs, older than those with aggressive BLPDs, had lower frequencies of Th‐naive cells and higher frequencies of Th‐M cells than those in aggressive BLPD patients who were younger (Supplementary figure 3). For all other T‐cell signatures, we did not observe any statistically significant association with age in the healthy control (HC) group (Supplementary figures 2b, 3 and 4), so those signatures were treated as age‐independent variables and consequently not affected by the age difference between indolent and aggressive BLPD groups.

**Figure 1:**
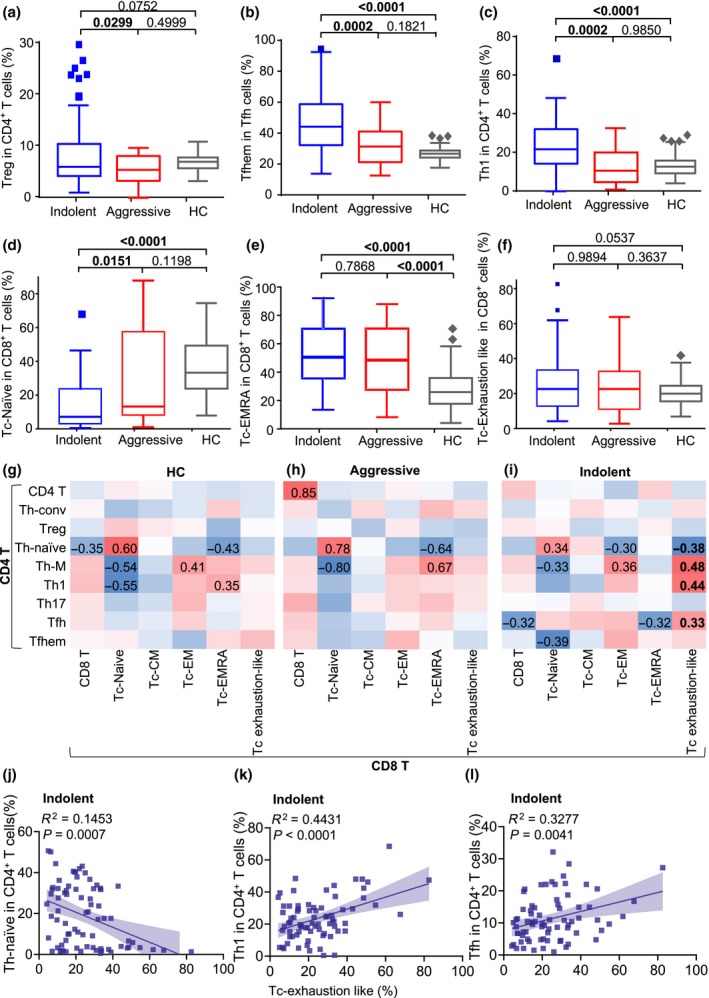
Different T‐cell immunological signatures among indolent BLPDs, aggressive BLPDs and HCs. PBMCs from patients with indolent BLPDs (*n* = 75, blue squares) or aggressive BLPDs (*n* = 19, red squares) and HC (*n* = 66, grey diamonds) were analysed for T‐cell subsets by flow cytometry as in Supplementary figure 1. **(a–f)** The frequencies of Treg in total CD4^+^ T cells **(a)**, Tfhem in total Tfh cells **(b)**, Th1 in total CD4^+^ T cells **(c)**, Tc‐naïve in total CD8^+^ cells **(d)**, Tc‐EMRA in total CD8^+^ cells **(e)** and Tc exhaustion‐like cells in total CD8^+^ cells **(f)** are shown as box‐and‐whisker plots for group comparisons. **(g–i)** Correlation matrix showing the correlations between the frequencies of CD4^+^ T‐cell subsets and CD8^+^ T‐cell subsets in HC individuals **(g)**, aggressive **(h)** and indolent (i) BLPD patients, respectively. The cells with *P*‐values < 0.01 were marked with the Pearson r values. **(j–l)** The frequencies of Th‐naïve **(j)**, Th1 **(k)** and Tfh **(l)** in total CD4^+^ T cells are shown as dot plots for the associations with Tc exhaustion‐like in total CD8^+^ T cells in indolent individuals. Flow cytometric analysis was performed without technical replication. Nonparametric Mann–Whitney *U*‐tests in **(a–f)** and linear regression correlations in **(g–l)**. *P*‐values < 0.05 were considered statistically significant.

Within CD4^+^ Th cells, we detected a mild increase of regulatory T (Treg) cells in indolent BLPDs than those in aggressive BLPDs and HCs (Figure 1a), in line with a previous report showing the upregulation of Treg cells in CLL patients.^11^ Tfh cells are instrumental to support B‐cell effector differentiation and function, in particular sustaining germinal centre B cells for affinity maturation and memory formation.^12^ The high frequency of CXCR5^+^CCR7^low^PD‐1^high^ effector memory follicular helper T (Tfhem) cells in total CXCR5^+^ Tfh cells indicates an excessive activation of Tfh cells.^13^ A previous study showed the help signals CD40L and IL‐4 from Tfh cells could help FL cells for survival and growth.^14^ We observed a striking upregulation of the frequencies of Tfhem cells in indolent BLPDs, as compared to aggressive BLPDs and HCs (Figure 1b), suggesting that the aberrant Tfh cell‐mediated help might be a common mechanism underlying the survival and proliferation of indolent BLPDs. Another Th functional subset, CXCR3^+^CCR6^−^ Th1 cells, also elevated significantly in indolent BLPD patients but not in aggressive BLPD patients (Figure 1c).

CD8^+^ T cells play a major role in preventing the development of BLPDs^2^. We observed a vast reduction of CD45RA^+^CCR7^+^ Tc‐naïve cells in indolent BLPDs (Figure 1d) but a significant increase of CD45RA^+^CCR7^−^ terminally differentiated effector memory Tc cells (Tc‐EMRA) in both indolent and aggressive BLPDs (Figure 1e). There was a trend of increase, though not statistically significant (*P* = 0.0537), for the frequencies of CCR7^−^PD‐1^+^ Tc cells, a pool of effector memory Tc cells expressing exhaustion marker PD‐1^15^ (designated as Tc exhaustion‐like) in indolent BLPD patients (Figure 1f). Therefore, Tc cells were highly activated and likely driven into exhaustion especially in indolent BLPDs.

We next tested the potential interactions between Th and Tc cells by examining the association between Th and Tc signatures. In HCs and BLPDs, Th‐ and Tc‐naïve subsets positively correlated with each other (Figure 1g and h). In addition, Th‐naïve subset negatively correlated with Tc‐EMRA or Tc‐EM subsets and vice versa (Figures 1g and h). These correlations indicate the synchronised activation between Th and Tc cells in healthy and aggressive BLPDs. Intriguingly, indolent BLPDs demonstrated a series of new correlations between Th subsets and Tc exhaustion‐like subset (Figure 1i). Tc exhaustion‐like negatively correlated with Th‐naïve (Figure 1j) but positively correlated Th1 and Tfh cells (Figures 1k and l). Th1 cells promote the effector function of Tc cells,^16^ suggesting that the Th1‐driven effector differentiation of CD8^+^ T cells towards exhaustion is a specific feature of indolent BLPDs. The correlation between Tfh differentiation and Tc exhaustion is likely explained by the fact that both differentiation processes are driven by chronic antigen stimulation.^17^, ^18^ Collectively, T‐cell phenotypes in indolent but not aggressive BLPDs showed a lymphomagenesis‐favourable immunological environment including enhanced immunosuppression from Treg cells, boosted B helper from Tfh cells and elevated Th1‐driven exhaustion of Tc cells.

### T‐cell signatures stratify BLPDs

Since T‐cell immune signatures were found particularly associated with indolent BLPDs, we then hypothesised that certain T‐cell signatures might selectively mark indolent BLPDs. We first adopted principal component analysis (PCA) using all 15 T‐cell immune signatures generated by flow cytometry, but it was insufficient to efficiently separate aggressive vs. indolent groups (Supplementary figure 5a). The result could be explained by the heterogeneity for both indolent and aggressive groups which are composed of several subsets. The complexity might be further compounded by the fact that some signatures with marginal power for distinction could interfere with the clustering. Nevertheless, PC1 appeared to provide a better distinction than PC2 (Supplementary figure 5a), suggesting appreciable discriminative power of the major variables that contributed to PC1. Such variables with higher absolute loading values in PC1 were Th‐naïve/Th‐M, Tc‐naïve/Tc‐EM, Th1, Tfhem and Tc exhaustion‐like (Supplementary figures 5b and c).

**Figure 2:**
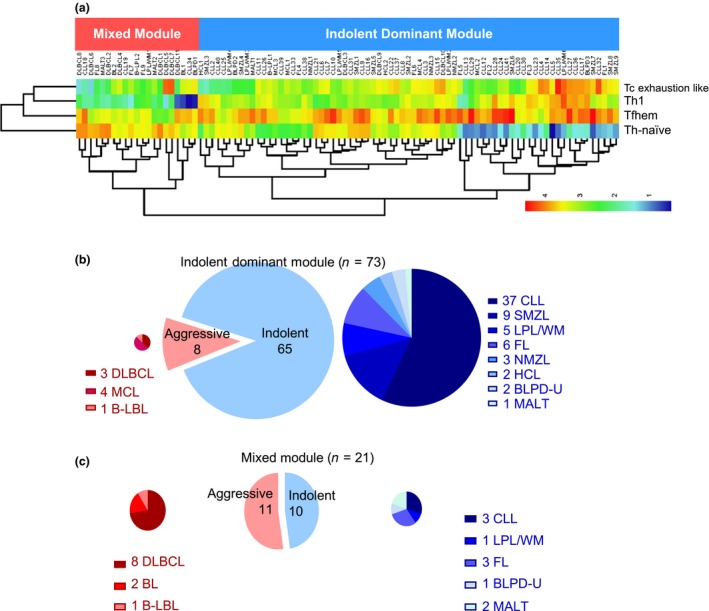
Prominent T‐cell immunological signatures stratify BLPDs and specifically associate with indolent BLPDs. **(a)** Top four variables (Th‐naïve, Tfhem, Th1 and Tc exhaustion‐like) from flow cytometric analysis were selected by random forest algorithm for the best binary classification of all 94 BLPD patients. Heatmap for the values of these four variables showing the clustering analysis of all 94 BLPD patients (the patients with their BLPD subtype labelled on the top of the heatmap). **(b, c)** Pie charts showing the compositions of patients classified as Indolent Dominant Module (*n* = 73, **b**) and as Mixed Module (*n* = 21, **c**).

Based on the promise, we then took a non‐hypothesis‐driven approach by applying a machine learning algorithm – random forest modelling to rank T‐cell immune signatures. This is a computationally extensive and robust data‐mining algorithm that accommodates a large set of variables to identify the most associated factors using an ensemble of classifications trees.^19^ Each of 15 T‐cell signatures (Supplementary figure 1) was regarded as one variable. The random forest algorithm selected Th‐naïve, Tfhem, Th1 and Tc exhaustion‐like as the top‐ranked variables to generate a binary classification of BLPD patients (Figure 2a). One group, marked by a T‐cell signature of low frequencies for Th‐naïve cells and high frequencies for Th1, Tfhem and Tc exhaustion‐like cells, was predominantly composed of indolent BLPDs (65/73, 89% indolent BLPDs, Figure 2b), thus termed as the Indolent Dominant Module. The other group was named as the Mixed Module (10/21, 47.6% indolent and 11/21, 52.4% aggressive BLPDs, Figure 2c). Such binary classification generated by machine learning matched the clinical classifications for > 85% (65/75) indolent BLPDs but demonstrated little discriminative power for aggressive BLPDs, echoing the conclusion drawn from the comparative analysis between indolent and aggressive groups that dysregulated T‐cell signatures were predominantly identified in indolent but not aggressive BLPDs (Figure 1).

### T‐cell signatures predict chemotherapy response and survival in indolent BLPDs

**Figure 3:**
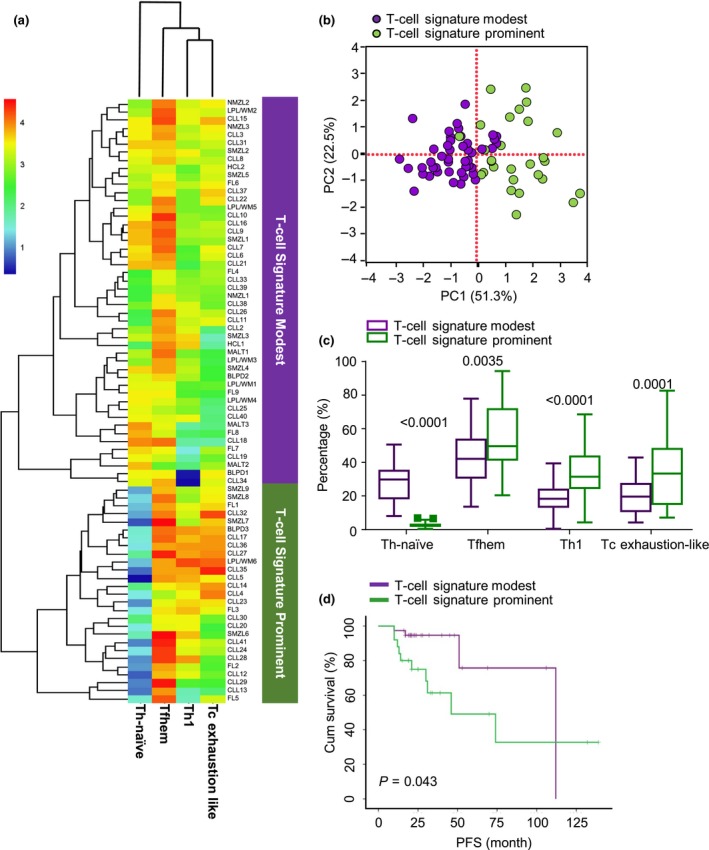
Prominent T‐cell immunological signatures predict chemotherapy response and prognosis of indolent BLPDs. **(a)** Heatmap for the values of top four variables (Th‐naïve, Tfhem, Th1 and Tc exhaustion‐like) showing the clustering analysis of all 75 indolent BLPD patients. **(b)** Top four variable‐based PCA showing the distribution of all indolent BLPD patients (*n* = 75). Green: the T‐cell Signature Prominent signature group (*n* = 27); Purple: the T‐cell Signature Modest signature group (*n* = 48). **(c)** Box‐and‐whisker plots (traditional Turkey whiskers) showing comparisons between T‐cell Signature Prominent and T‐cell Signature Modest groups for the frequencies of Th‐naïve, Tfhem, Th1 and Tc exhaustion‐like cell subsets. **(d)** Progression‐free survival (PFS) curves for T‐cell Signature Prominent vs. T‐cell Signature Modest group patients, calculated by using the Kaplan–Meier method. Nonparametric Mann–Whitney *U*‐tests in **(c)** and univariate analysis (the log‐rank test) in **(d)**. *P*‐values < 0.05 were considered statistically significant.

Since T‐cell immune signatures were specifically associated with indolent BLPDs, we next investigated the relationship between T‐cell signatures and disease prognosis in indolent BLPDs. Using the top four variables identified by random forest modelling (Figure 2a), 75 indolent BLPD patients were clustered into two groups (Figures 3a and b). Patients in one group displayed a more prominent immune signature of T cells (T‐cell Signature Prominent, *n* = 27) including significantly lower frequencies of Th‐naïve cells and higher frequencies of Th1, Tfhem and Tc exhaustion‐like cells than these variables of patients in the other group (T‐cell Signature Modest, *n* = 48) (Figure 3c). By comparing the clinical data between two groups (Table 1), we found patients with prominent T‐cell signatures (T‐cell Signature Prominent group) demonstrated worse prognosis than those with modest T‐cell signatures (T‐cell Signature Modest group), indicated by reduced 3‐year progression‐free survival (PFS) rates (61.4 ± 11.4% vs. 94.7 ± 3.7%, *P* = 0.043) (Figure 3d). Consistent with reduced PFS rates, the rate of elevated serum lactate dehydrogenase (LDH) (80.0% vs. 40.0%, *P* = 0.032) and the deletion of 17p (18.2% vs. 5.1%, *P* = 0.100), usually associated with poorer prognosis, were higher in the T‐cell Signature Prominent group than in the T‐cell Signature Modest group. In addition, patients in the T‐cell Signature Prominent group showed a lower response rate to immuno‐chemotherapies than the T‐cell Signature Modest group (CR rate 23.8% vs. 50.0%, *P* = 0.035, Table 1).

**Table 1:** The comparison of the clinical characteristics between the two modules inside indolent BLPD patients

	T‐cell signature modest (*N* = 48)	T‐cell signature prominent (*N* = 27)	*P*‐value
Del13q			
Yes	3/35 (8.6%)	2/20 (10.0%)	0.859
No	32/35 (91.4%)	18/20 (90.0%)	
Trisomy 12			
Yes	7/31 (22.6%)	4/18 (22.2%)	0.977
No	24/31 (77.4%)	14/18 (77.8%)	
Del11q			
Yes	2/24 (8.3%)	0/14 (0%)	0.267
No	22/24 (91.7%)	14/14 (100%)	
Del17p			
Yes	2/39 (5.1%)	4/22 (18.2%)	0.100
No	37/39 (92.5%)	18/22 (81.8%)	
Complicated karyotype			
Yes	4/38 (10.5%)	5/24 (20.8%)	0.262
No	34/38 (89.5%)	19/24 (79.2%)	
Elevated LDH			
Yes	10/25 (40.0%)	8/10 (80.0%)	0.032*
No	15/25 (60.0%)	2/10 (20.0%)	
Chemotherapy response			
CR	16 (50.0%)	5 (23.8%)	0.035*
PR	14 (43.8%)	9 (42.9%)	
SD	0 (0%)	3 (14.3%)	
PD	2 (6.2%)	4 (19.0%)	
Progression‐free survival (3 years)	94.7 ± 3.7%	61.4 ± 11.4%	0.043*
Overall survival (3 years)	97.1 ± 2.9%	77.2 ± 10.6%	0.122

Complicated karyotype: more than or equal to 3 chromosomal aberrations. CR, complete remission; Del, deletion; LDH, lactate dehydrogenase; PD, progress disease; PR, partial remission; SD, stable disease.

* *P* < 0.05.

## Discussion

The dysregulation of T‐cell function was reported in individual subtypes of BLPDs, but the overall picture has not been depicted. Our comprehensive analysis of all major subsets of both CD4^+^ and CD8^+^ T cells included a broad range of aggressive and indolent BLPDs, thus fulfilling this gap. Our results suggested that the dysregulation of T‐cell function was particularly associated with indolent rather than aggressive BLPDs. First, the immunophenotyping demonstrated that the frequencies of major CD4^+^ and CD8^+^ T‐cell functional subsets in aggressive BLPDs were largely comparable to those in HCs whereas indolent BLPDs showed the increase in Treg, Th1 and Tfhem and the decrease in Tc‐naïve subsets compared to both aggressive BLPDs and HCs. Second, the random forest modelling unbiasedly selected Th‐naïve, Th1, Tfhem and Tc exhaustion‐like as the top‐ranked variables to generate a binary classification of all BLPD patients, with > 85% indolent patients enriched in one group.

The altered function of T cells likely constitutes a favourable immune environment for the development and progress of indolent BLPDs. Enhanced Tfh function, indicated by the increased frequencies of Tfhem cells, may support malignant B cells to survive and growth via Tfh effector molecules such as CD40L and IL‐21.^12^ Th1 cells promote CD8^+^ T cell to differentiate into effectors which can gradually acquire the state of exhaustion by the chronic stimulation of malignant B cells.^18^ Furthermore, since IFN‐γ produced by Th1 cells can upregulate PD‐L1 expression on tumour cells,^16^ it is conceivable that the excessive Th1 differentiation may also render the risk of CD8^+^ T‐cell exhaustion through the PD‐1‐PD‐L1 interactions. Treg cells were found upregulated in indolent compared to aggressive BLPDs, indicating a greater immune suppression in indolent BLPDs, which might cripple the effector differentiation and killing function of Tc cells. Therefore, the development of indolent BLPDs was accompanied by an immune environment not only promoting B‐cell growth by increased Tfh function but also impairing immune surveillance due to enhanced Treg‐mediated immune suppression and exhaustion‐associated dysfunction of Tc cells (Supplementary figure 6).

Notably, the dysregulated T‐cell immune signatures associated with indolent BLPDs were more prominent in a subgroup (T‐cell Signature Prominent) that showed special tumour characteristics and poor prognosis. Patients in this T‐cell Signature Prominent subgroup, compared to the other subgroup with modest signature of T‐cell dysregulation (T‐cell Signature Modest), demonstrated elevated LDH which indicates high tumour load and the higher frequencies of del17p which serves as a crucial genetic marker for aggressive behaviour of tumour cells.^20^ It is difficult to distinguish whether the stronger dysregulation of T‐cell immunity was the cause or the consequence of the features for malignancy although the worse prognosis in the T‐cell Signature Prominent subgroup suggests the impaired immune surveillance may lead to the progress of indolent BLPDs. One way to examine this notion is to test whether the reinstatement of normal T‐cell function can benefit the treatment of indolent BLPDs. Lenalidomide is an approved therapy with immunomodulatory functions. It was reported to improve the impaired T‐cell immune synapse in FL^21^ and CLL,^6^ to enhance the activation of CD8^+^ T and NK cells, and to reduce Treg cells and rebalance Th subsets.^22^ The BTK inhibitor ibrutinib is another candidate to help the immune normalisation, which was recently demonstrated to improve the function of T cells including modulating Th1/Th2 subsets.^23^ Future studies are required to examine whether patients with superior responses to lenalidomide, ibrutinib or other immunomodulatory drugs demonstrate better improvement of T‐cell immunity. Complementary approach is to utilise mouse models to test whether the prone differentiation to Tc exhaustion or the excessive help of Tfh cells can promote the development of BLPDs.

Our observations also shed light on the application of immunotherapies for BLPDs. For example, immune checkpoint inhibition has been trialled as a new therapy for BLPDs. Although > 60% objective response rates (ORRs) were recorded for PD‐1 blockade in Hodgkin lymphoma, clinical outcomes with PD‐1 blockade in BLPDs (non‐Hodgkin's lymphoma) have been disappointing, with ORRs as ~ 10% or less.^24^ The results from our study suggest indolent BLPDs might be more suitable for PD‐1 blockage therapy, particularly those with prominent signatures for dysregulated T‐cell immunity including the exhaustion of Tc cells. Nevertheless, multiple defects of T‐cell function revealed by our study suggest that a combination strategy to target not only Tc cells but also Tfh, Th1 and Treg cells likely yields more pronounced outcomes.

Normalisation of immune function in cancer can benefit patients by not only improving the efficacy of immunotherapies but also preventing relapses.^25^ The identification of dysregulated T‐cell immunity in indolent BLPDs and the association between prominent immune signatures and poor prognosis provide rationales to design new strategies for immune normalisation in BLPDs. Future studies are warranted to validate aberrant T‐cell immune signatures in larger cohorts of indolent BLPDs and further dissect the subtypes of indolent BLPDs that are more susceptible to the dysregulation of T‐cell function and more responsive to immunotherapies.

## Methods

### Patient samples

Treatment‐naïve patients of BLPD (non‐Hodgkin's lymphoma) aged from 15 to 81 (*n* = 94) were enrolled into this study. The diagnosis was according to the 4th edition of World Health Organization Classification.^1^ At the time of sampling, patients diagnosed with infection or autoimmune diseases were excluded from this study. Peripheral blood samples were collected in Blood Diseases Hospital, Tianjin, China. Written informed consents were signed in accordance with the requirements of the Declaration of Helsinki. Blood samples from healthy individuals aged from 25 to 50 (*n* = 66) were collected as controls. The Ethics Committee of Blood Disease Hospital (NI2016001‐EC‐1) approved this study. The peripheral blood mononuclear cells (PBMCs) were isolated by density gradient centrifugation using Ficoll‐Paque (GE Healthcare Bio‐sciences AB, Uppsala, Sweden), resuspended in 10% DMSO (Sigma Aldrich, Saint Louis, MO, USA) and 90% foetal bovine serum (FBS; Gibco, Waltham, MA, USA) and stored at −80°C until use. All the clinical data were collected at diagnosis.

### Flow cytometric analysis for immune phenotyping

Peripheral blood mononuclear cells were thawed in warm media, washed twice and resuspended at 1 × 10^7^ viable cells per mL. About 50–100 μL cells per well were stained for 45 min at room temperature with antibodies listed in Supplementary table 2. Cells were washed three times with FACS buffer (PBS supplemented with 2% FBS and 0.1% EDTA) and resuspended in 200 μL FACS buffer. A total of 100 000 lymphocytes per sample were collected using DIVA 6.0 software on a FACS Aria III Cytometer (BD Biosciences, Franklin Lakes, NJ, USA). Data analysis was performed using FlowJo v9 software (Tree Star, Inc., Ashland, OR, USA). The detailed gating strategy for T‐cell subsets is outlined in Supplementary figure 1.

### Statistical analysis

Prism software (version 7.0; GraphPad Software) was used for statistical analysis of flow cytometry data. No randomisation or exclusion of data points was used. Nonparametric Mann–Whitney *U*‐tests were used for unpaired comparisons between groups. Linear regression correlations of ages and percentages of individual T‐cell subset were calculated in each group. Progression‐free survival (PFS) was calculated from the diagnosis to the disease progression or last follow‐up. For the survival patients, the median follow‐up time is 22 months (13–139 months). Fisher's exact test or the chi‐square test of two‐sided was used to determine the statistically significant differences for the clinical and immunological characteristics between two groups. Survival curves were constructed using the Kaplan–Meier method, and prognostic features were evaluated by univariate analysis (i.e. the log‐rank test) using the SPSS Statistical Software Package (version 13.0; SPSS Inc., Chicago, IL. USA). *P*‐values < 0.05 were considered statistically significant.

### Principal component analyses

To visualise immune‐phenotyping data and explore correlations between variables, we used principal component analysis (PCA). PCA statistically aggregates variables, reduces the number of observed variables into smaller number of principal components and reduces the dimensionality of the immune‐phenotyping data. The PCAs were conducted using JMP 11.0 software.

### Variable ranking and clustering analysis

Random forest algorithm (as contained within the R package ‘randomForestSRC’ version 4.6–7; R version 3.3.0) was used to rank the importance of variables (the frequencies of T‐cell subsets).^19^ All 15 variables used in analysis were shown in Supplementary table 3. Heatmap algorithm (‘pheatmap’ version 4.6–7 in R version 3.3.0) was used to generate nonsupervised clustering analysis,^26^ based on top‐ranked variables.

## Conflict of interest

The authors declare no conflict of interest.
