# Supplementary material for: Prominent immune signatures of T cells are specifically associated with indolent B‐cell lymphoproliferative disorders and predict prognosis
Source: Clin Transl Immunology. 2020 Jan 22;9(1):e01105. doi: 10.1002/cti2.1105 (PMC6975127; doi:10.1002/cti2.1105)
Supplement: Supplementary file 1 [file CTI2-9-e01105-s001.pdf]

Figure S1

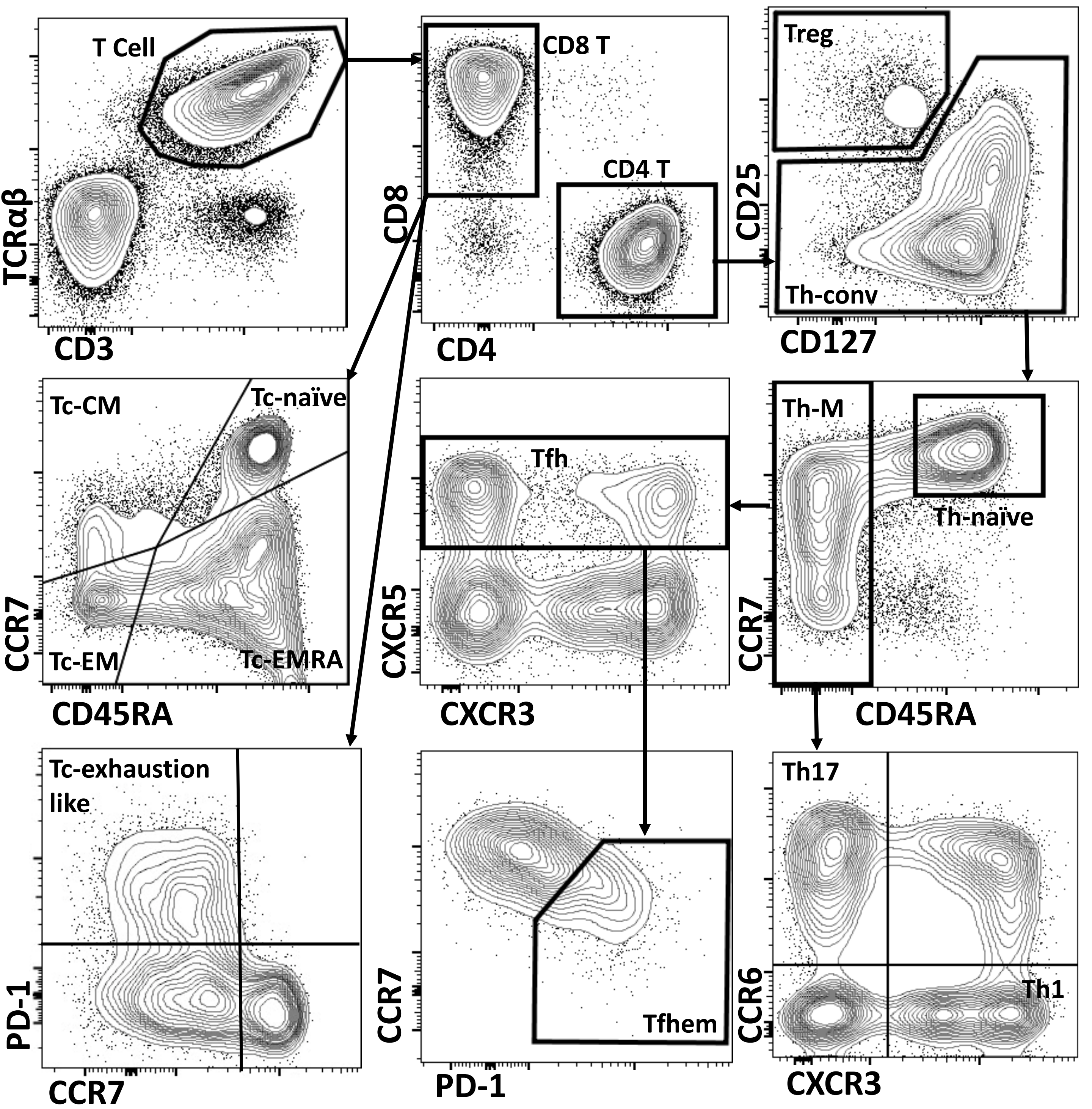

Figure S1. Phenotypic characterization of T cell subsets by flow cytometry. Each marked T cell subse was recognized as a immunological signature.

Figure S2

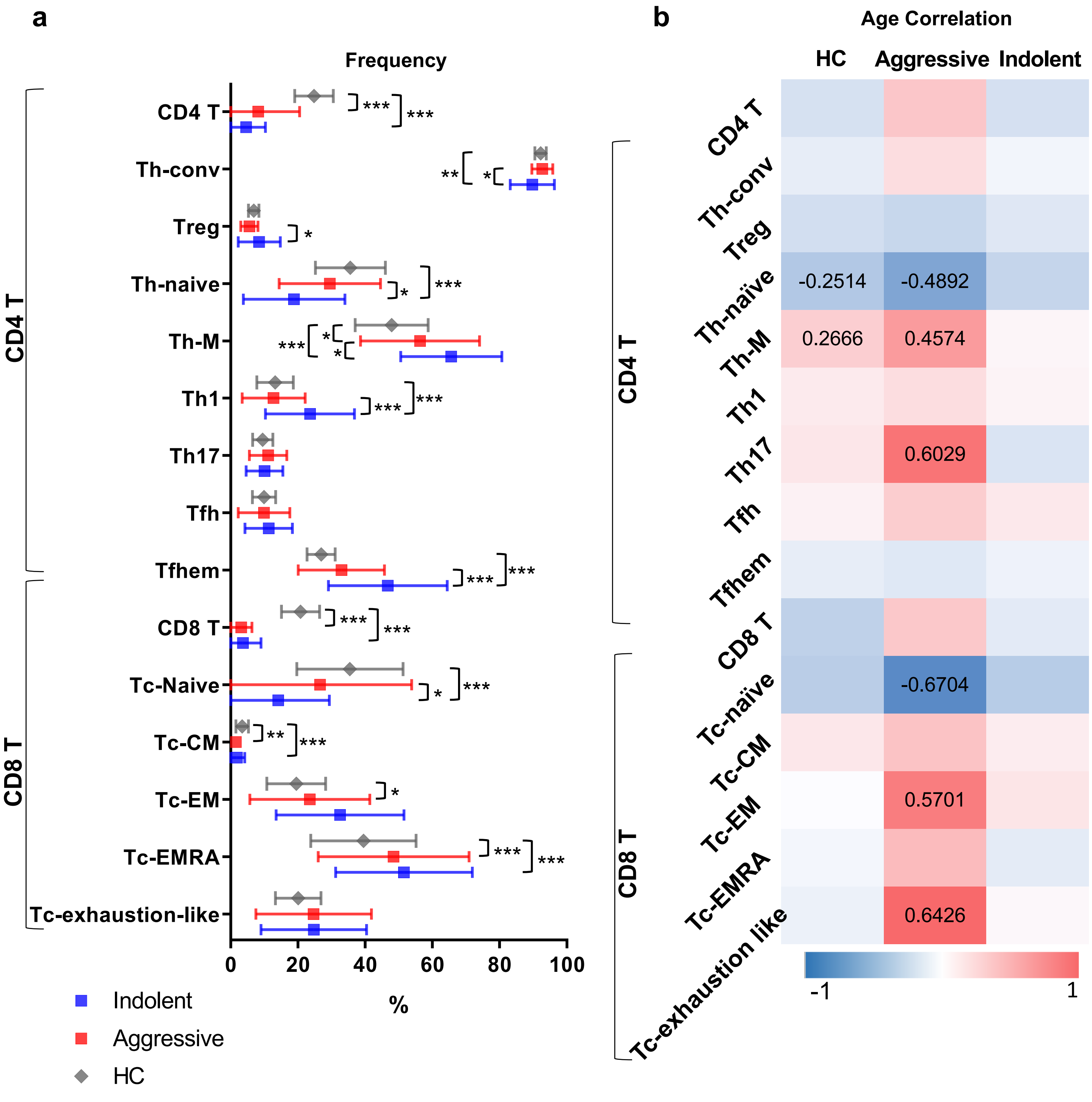

Figure S2. Immunological status of different CD4⁺ T cell and CD8⁺ T cell subsets in BLPD patients. The frequency (**a**) and age correlation (**b**) of each T cell immunological signatures in Indolent group, Aggressive group and healthy control group. *P*-values in (**a**): * $P<0.05$, ** $P < 0.01$, *** $P < 0.001$. The cells in (**b**) with *P*-value < 0.05 were marked with the Pearson r values.

Figure S3

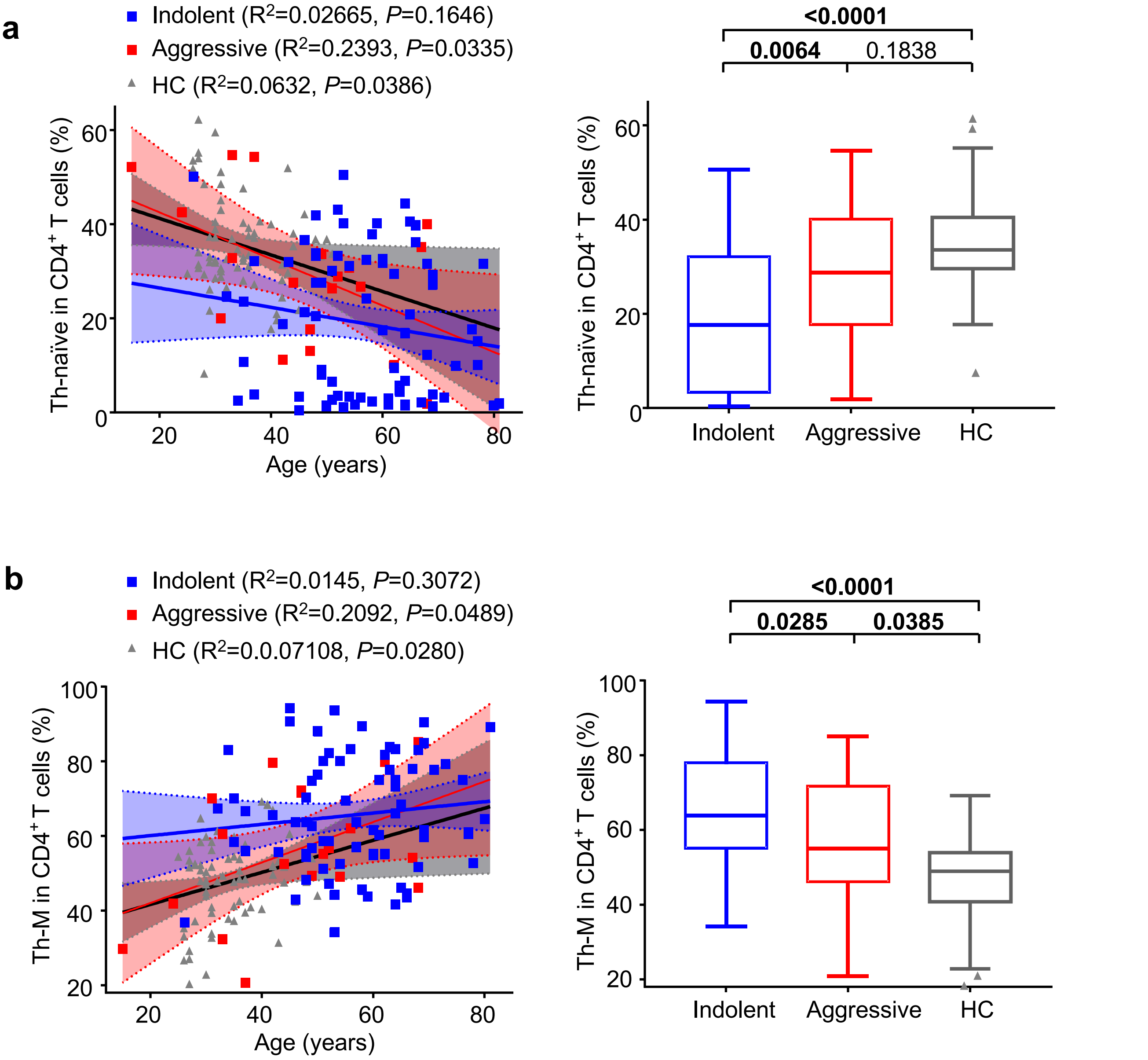

Figure S3. Immunological status of different CD4⁺ T cell subsets in BLPD patients.

The age correlation and frequency of (**a**) Th-naïve and (**b**) Th-M in indolent BLPD patients, aggressive BLPD patients and HC groups.

Figure S4

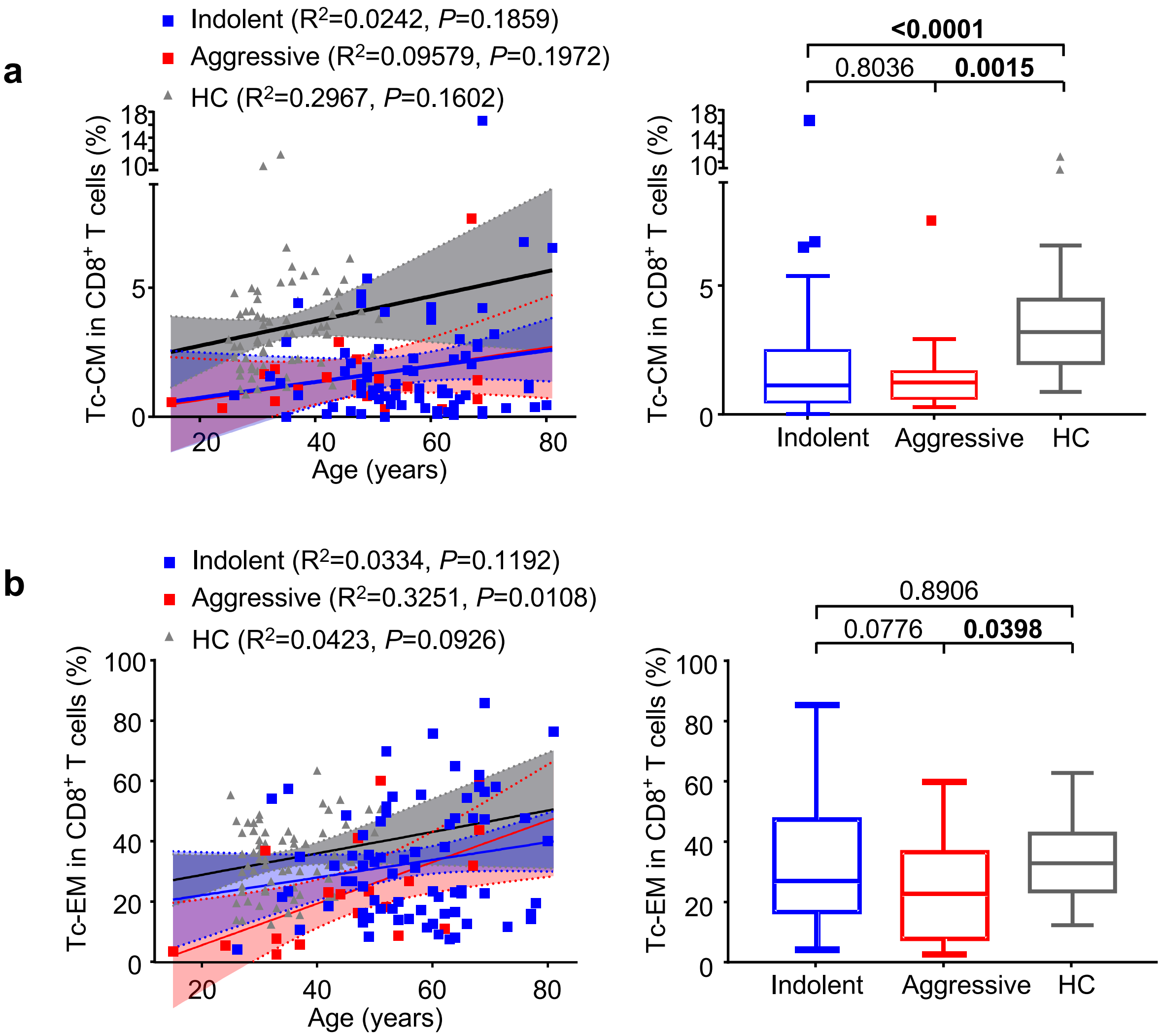

Figure S4. Immunological status of different CD8$^+$ T cell subsets in BLPD patients.
The age correlation and frequency of (**a**) TcCM and (**b**) TcEM cells in indolent BLPD patients, aggressive BLPD patients and HC groups.

Figure S5

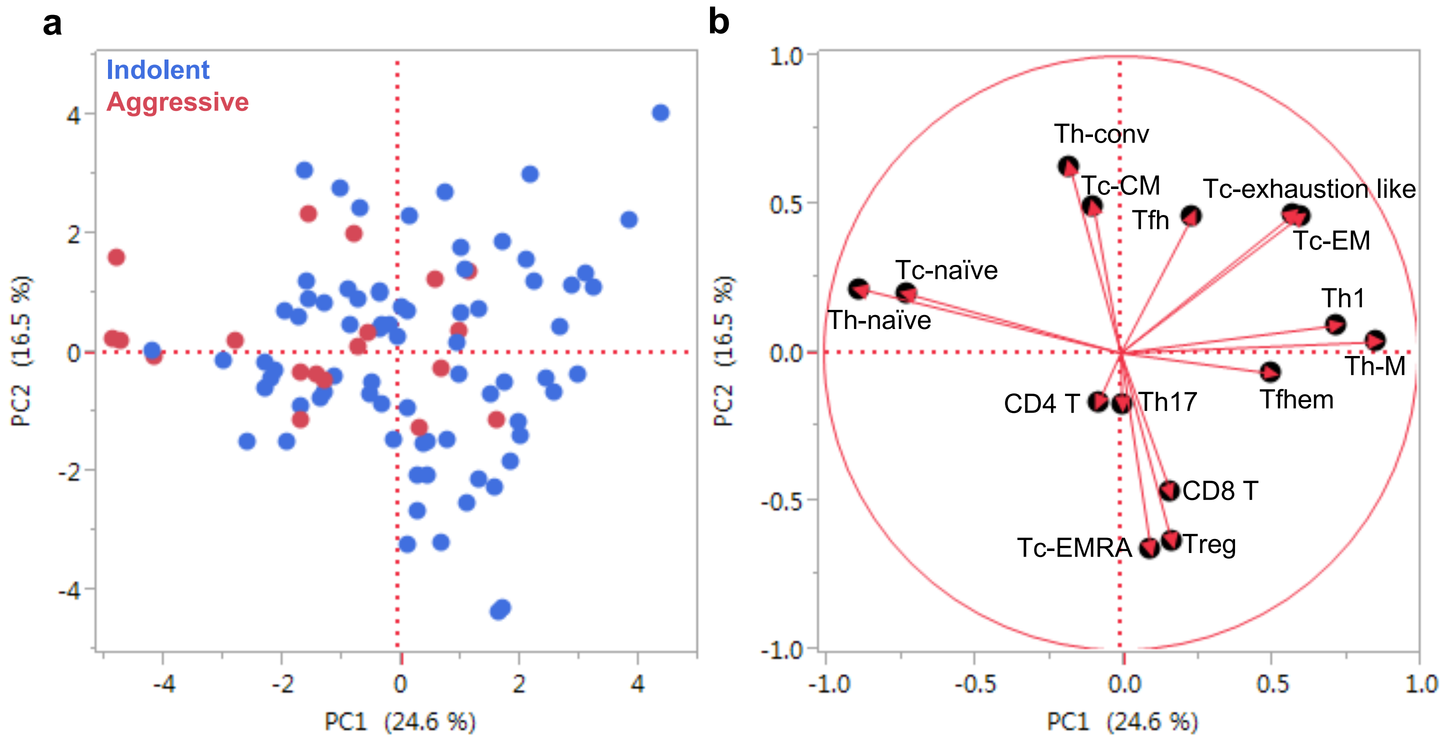

c

	PC1	PC2
**CD4 T**	-0.0754	-0.1666
**Th-conv**	-0.1701	**0.6242**
**Treg**	0.1718	**-0.6362**
**Th-M**	**0.8621**	0.0374
**Th1**	**0.7291**	0.0933
**Th17**	0.0041	-0.1727
**Th-naïve**	**-0.8780**	0.2162
**Tfhem**	**0.5064**	-0.0686
**Tfh**	0.2401	**0.4571**
**CD8 T**	0.1664	**-0.4646**
**Tc-CM**	-0.0915	**0.4912**
**Tc-EM**	**0.6068**	**0.4624**
**Tc-EMRA**	0.1013	**-0.6580**
**Tc-naïve**	**-0.7172**	0.1991
**Tc-exhaustion-like**	**0.5795**	**0.4646**

Figure S5. Principal components analysis (PCA) analysis of all patients utilizing all 15 immunological signatures. (**a**) The clustering of all patients including indolent BLPDs (n = 75, blue circles) or aggress BLPDs (n = 19, red circles) based on all 15 immunological signatures. The loading plot (**b**) and factor loading matrix (**c**) of all 15 immunological signatures. The numbers labeled in (**c**) represent the contribution of each immunological signatures for each components.

Figure S6

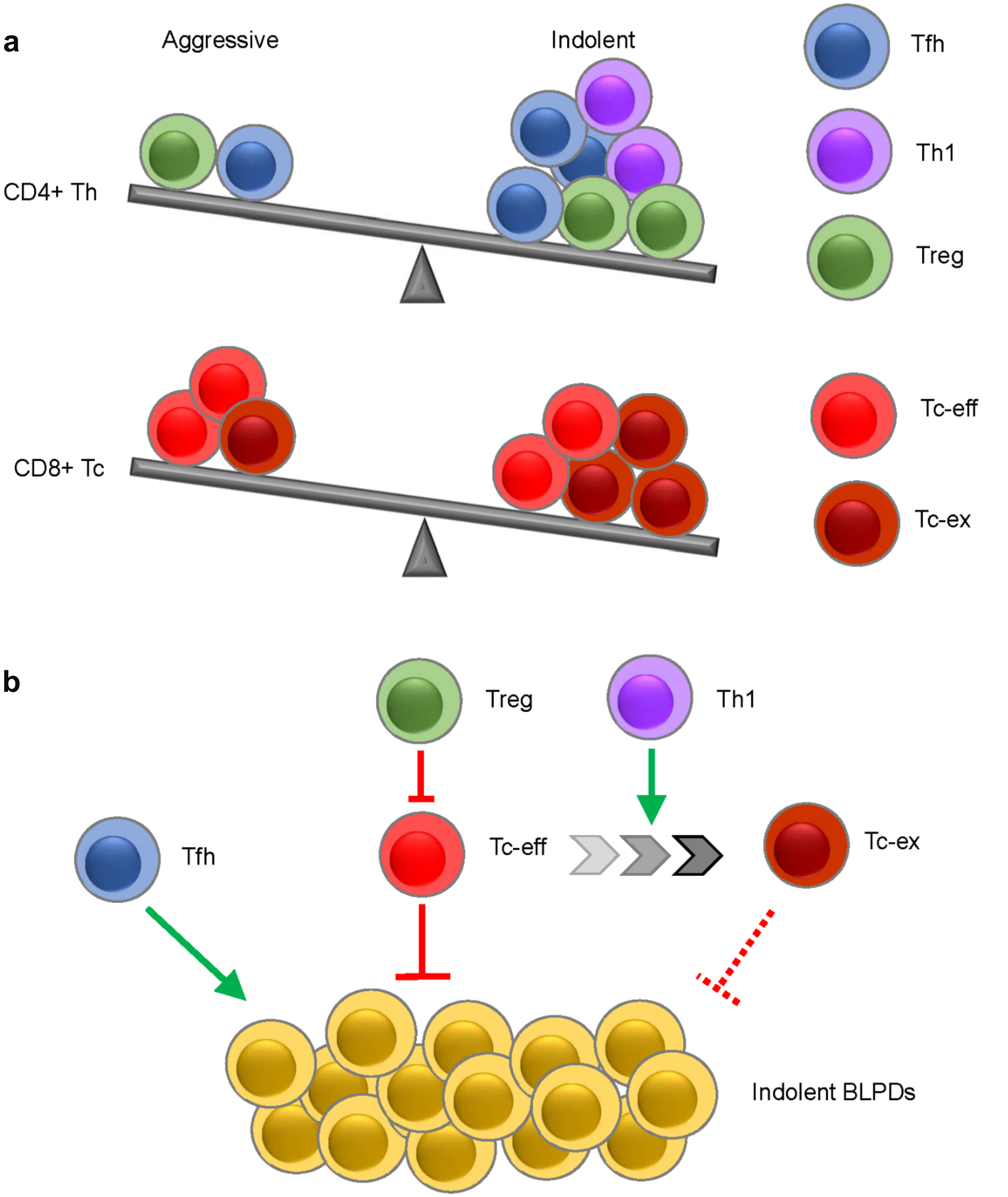

Figure S6. Schematic of dysregulated T-cell immunity in indolent BLPDs.

(**a**) The dysregulated CD4^{+} Th and CD8^{+} Tc subsets in indolent BLPDs. (**b**) The proposed model for the dysregulated T-cell function underlying the development of indolent BLPDs. 1) Increased Tfh cells promote lymphoma cells to growth; 2) Enhanced Treg function suppresses the immune surveillance of effector Tc (Tc-eff) cells; 3) Increased Th1 cells drive Tc activation and differentiate towards exhaustion (Tc-ex), which impairs immune surveillance.
