# Supplementary material for: Prominent immune signatures of T cells are specifically associated with indolent B‐cell lymphoproliferative disorders and predict prognosis
Source: Clin Transl Immunology. 2020 Jan 22;9(1):e01105. doi: 10.1002/cti2.1105 (PMC6975127; doi:10.1002/cti2.1105)
Supplement: Supplementary file 2 [file CTI2-9-e01105-s002.pdf]

Supplementary Tables

Table S1: Clinical characteristics of BLPD patients.

	**Aggressive BLPD patients**					**Indolent BLPD patients**									**Health Control**
**Subtype**	Total	DLBCL	BL	MCL	B-ALL	Total	CLL /SLL	NMZL	SMZL	LPL/WM	MALT	BLPD-U	HCL	FL	
**Number**	19	11	2	4	2	75	40	3	9	6	3	3	2	9	66
**Age, year: median (range)**	47 (15-68)	47 (15-68)	31 (31-33)	56.5 (47-68)	(42-54)	56 (26-81)	59 (42-80)	65 (57-73)	60 (32-81)	62 (51-69)	46 (26-53)	49 (45-69)	(49-57)	37 (34-64)	32.5 (25-50)
**Sex (Female/Male)**	4/14	2/9	1/1	1/3	0/2	27/52	13/24	1/2	4/5	1/5	3/0	0/3	1/1	3/6	40/26

Table S2. Flow cytometric antibodies used in flow cytometry analysis.

Antigen	Clone	Fluorochrome	Vendor
TCRαβ	IP26	FITC	Biolegend
CD3	UCHT1	Brilliant Violet 650	BD Biosciences
CD4	RPA-T4	Alexa Fluor 700	Biolegend
CD8	RPA-T4	Brilliant Violet 510	Biolegend
CD25	M-A251	PE-CF594	BD Biosciences
CD127	A019D5	Brilliant Violet 605	Biolegend
CD45RA	HI100	APC-Cy7	Biolegend
CXCR3	G025H7	Brilliant Violet 421	Biolegend
CCR6	G034E3	PE	Biolegend
CCR7	G043H7	PE-Cy7	Biolegend
CXCR5	RF8B2	Alexa Fluor 647	BD Biosciences
PD-1	EH12.2H7	Brilliant Violet 711	Biolegend

Table S3. T cell subset variables used in variables selection and clustering analysis.

T cell subset	Abbreviation	Cell marker
$CD4^+$ T cells	CD4 T	TCRαβ$^+$ $CD3^+$ $CD4^+$
T conventional cells	Th-conv	TCRαβ$^+$ $CD3^+$ $CD4^+$ $CD127^{low-high}$ $CD25^{neg-low}$
Regulatory T cells	Treg	TCRαβ$^+$ $CD3^+$ $CD4^+$ $CD127^{low}$ $CD25^{high}$
Naïve helper T cells	Th-naïve	TCRαβ$^+$ $CD3^+$ $CD4^+$ $CD127^{low-high}$ $CD25^{neg-low}$ $CD45RA^{high}$ $CCR7^+$
Effector-memory T cells	Th-EM	TCRαβ$^+$ $CD3^+$ $CD4^+$ $CD127^{low-high}$ $CD25^{neg-low}$ $CD45RA^{low}$ $CCR7^{low-high}$
Type 1 helper T cells	Th1	TCRαβ$^+$ $CD3^+$ $CD4^+$ $CD127^{low-high}$ $CD25^{neg-low}$ $CD45RA^{low}$ $CCR7^{low-high}$ $CXCR3^+$ $CCR6^-$
Type 17 helper T cells	Th17	TCRαβ$^+$ $CD3^+$ $CD4^+$ $CD127^{low-high}$ $CD25^{neg-low}$ $CD45RA^{low}$ $CCR7^{low-high}$ $CCR6^+$ $CXCR3^-$
Follicular helper T cells	Tfh	TCRαβ$^+$ $CD3^+$ $CD4^+$ $CD127^{low-high}$ $CD25^{neg-low}$ $CD45RA^{low}$ $CXCR5^+$
Effector-memory follicular helper T cells	Tfhem	TCRαβ$^+$ $CD3^+$ $CD4^+$ $CD127^{low-high}$ $CD25^{neg-low}$ $CD45RA^{low}$ $CXCR5^+$ $CCR7^{low}$ PD-1^{high}
$CD8^+$ T cells	CD8 T	TCRαβ$^+$ $CD3^+$ $CD8^+$
Naïve $CD8^+$ T cells	Tc-naïve	TCRαβ$^+$ $CD3^+$ $CD8^+$ $CD45RA^{high}$ $CCR7^{high}$
Central memory $CD8^+$ T cells	Tc-CM	TCRαβ$^+$ $CD3^+$ $CD8^+$ $CD45RA^{low}$ $CCR7^{high}$
Effector memory $CD8^+$ T cells	Tc-EM	TCRαβ$^+$ $CD3^+$ $CD8^+$ $CD45RA^{high}$ $CCR7^{low}$
$CD45RA^{high}$ effector memory $CD8^+$ T cells	Tc-EMRA	TCRαβ$^+$ $CD3^+$ $CD8^+$ $CD45RA^{high}$ $CCR7^{low}$
PD-1^+ $CCR7^-$ $CD8^+$ T cells	Tc-exhaustion-like	TCRαβ$^+$ $CD3^+$ $CD8^+$ $CCR7^{low}$ PD-1^{high}
